# Neuromuscular Diseases Affect Number Representation and Processing: An Exploratory Study

**DOI:** 10.3389/fpsyg.2021.697881

**Published:** 2021-09-06

**Authors:** Hendrikje Schmidt, Arianna Felisatti, Michael von Aster, Jürgen Wilbert, Arpad von Moers, Martin H. Fischer

**Affiliations:** ^1^Potsdam Embodied Cognition Group, Department of Psychology, University of Potsdam, Potsdam, Germany; ^2^Center for Special Educational and Psychological Needs, German Red Cross Hospitals Berlin, Berlin, Germany; ^3^Department of Inclusive Education, University of Potsdam, Potsdam, Germany; ^4^Department of Pediatrics, German Red Cross Hospitals Berlin, Berlin, Germany

**Keywords:** spatial-numerical associations, numerical processing, mathematics, child development, embodied cognition, neuromuscular disease, spinal muscular atrophy, Duchenne muscular dystrophy

## Abstract

Spinal muscular atrophy (SMA) and Duchenne muscular dystrophy (DMD) both are rare genetic neuromuscular diseases with progressive loss of motor ability. The neuromotor developmental course of those diseases is well documented. In contrast, there is only little evidence about characteristics of general and specific cognitive development. In both conditions the final motor outcome is characterized by an inability to move autonomously: children with SMA never accomplish independent motoric exploration of their environment, while children with DMD do but later lose this ability again. These profound differences in developmental pathways might affect cognitive development of SMA vs. DMD children, as cognition is shaped by individual motor experiences. DMD patients show impaired executive functions, working memory, and verbal IQ, whereas only motor ability seems to be impaired in SMA. Advanced cognitive capacity in SMA may serve as a compensatory mechanism for achieving in education, career progression, and social satisfaction. This study aimed to relate differences in basic numerical concepts and arithmetic achievement in SMA and DMD patients to differences in their motor development and resulting sensorimotor and environmental experiences. Horizontal and vertical spatial-numerical associations were explored in SMA/DMD children ranging between 6 and 12 years through the random number generation task. Furthermore, arithmetic skills as well as general cognitive ability were assessed. Groups differed in spatial number processing as well as in arithmetic and domain-general cognitive functions. Children with SMA showed no horizontal and even reversed vertical spatial-numerical associations. Children with DMD on the other hand revealed patterns in spatial numerical associations comparable to healthy developing children. From the embodied Cognition perspective, early sensorimotor experience does play a role in development of mental number representations. However, it remains open whether and how this becomes relevant for the acquisition of higher order cognitive and arithmetic skills.

## Introduction

Children born with neuromuscular degeneration are rare. Depending on the exact disease type, incidence rates between 1 and 5 out of 10,000 children have been reported in prevalence studies (for a review, see Deenen et al., 2015). Affected children tend to be looked at only deficit-oriented on their motor capacity. Both individual cognitive/psychosocial deficits and strengths might be neglected or not even detected in the first place. In particular, children and adolescents affected by spinal muscular atrophy (SMA) or Duchenne muscular dystrophy (DMD) suffer from loss of muscle strength and progressive motor impairments, with implications for daily physical, cognitive and social functioning (e.g., Billard et al., 1992). So far, cognitive consequences of inherited motor deficits have been explored through intelligence scales, such as Wechsler Intelligence Scales, Kaufman Assessment Battery, Standard and Progressive Matrices. These instruments cover verbal and non-verbal cognitive abilities and have revealed that the impact of the motor impairment on cognitive parameters depends on the genetic background and the specific neuromuscular disease, as described below.

In the present study, we will focus on one of these higher-level cognitive abilities, namely numerical cognition. Numeracy, the ability to comprehend and mentally manipulate quantitative non-symbolic and symbolic relations, is a key predictor for a healthy and successful life (Parsons and Bynner, 1997; Woloshin et al., 2001; Butterworth, 2018). In light of differences in arithmetic performances with respect to the two disease groups (Billard et al., 1992; von Gontard et al., 2002), we aim to answer the question of *how* numerical concepts are represented and processed in children with SMA and DMD. We begin with a brief summary of typical and atypical development of numerical cognition, followed by a description of these specific rare neuromuscular diseases, drawing attention to differences between the two medical conditions. Then we motivate the present study of numerical abilities in SMA and DMD from an embodied cognition perspective.

### Typical and Atypical Development of Numerical Cognition

Number processing, calculation and math reasoning constitute a complex cognitive domain that developmentally relies on certain core abilities to discriminate concrete non-symbolic magnitudes, that are available quite early in life. The achievement of further domain-specific cognitive representations and abilities in the numerical domain, like symbolic number processing (linguistic, Arabic), the spatially oriented mental number line, arithmetic procedures and mathematical reasoning abilities, is dependent on and accompanied by the development of domain-general abilities, like sensorimotor and visuospatial integration, working memory, language, attentional and affective regulation. Therefore, reasons, symptoms and course of atypical development of numerical cognition are manifold and diverse (Bachot et al., 2005; von Aster and Shalev, 2007; von Aster et al., 2021). As DMD and SMA are diseases with similar motor but quite different cognitive and social-emotional outcomes, it will be of interest how children of both groups differ according to their embodied cognitive numerical representations and developmental patterns of domain-specific and domain-general intellectual functioning.

### Spinal Muscular Atrophy

SMA is an autosomal recessive inherited disease. It is caused by mutations in the survival motor neuron1-gene (*SMN1*-gene) on chromosome 5. They result in less or total absence of the survival motor neuron-protein (SMN-protein) and only affect the anterior motor horn cell in the spinal cord. SMN-protein is essential for life, being necessary for information processing from the spinal cord to the muscles (Lefebvre et al., 1995). As a consequence, absence of the *SMN1*-gene results in general weakness with proximal muscles more affected than distal ones. Three types are distinguished in children and adolescents depending in the onset of the symptoms. Severity of disease depends on the amount of SMN-protein present and is negatively correlated with symptom onset (Calucho et al., 2018). If symptoms occur before adulthood, three types of SMA are distinguished: Children with SMA type 1 show symptoms within 6 months after birth; they will never be able to sit autonomously and their life expectancy without treatment is up to 2 years. In SMA type 2 symptoms occur after 6 months of age, allowing children to sit but never to stand or walk. Scoliosis is frequent and some patients need non-invasive ventilation. SMA type 3 is defined by sufficient muscle strength for walking without support and proximal weakness within the first two decades. People affected by SMA show normal sensory, cognitive, emotional and social functioning (Kolb and Kissel, 2015). Since 2017 and 2020 molecularly based medication has an essential impact on the course of the disease.

### Duchenne Muscular Dystrophy

DMD is an X-chromosomal recessive disorder caused by mutations on the short arm of the X-chromosome and therefore is exclusively affected in males. It results in the reduction of dystrophin protein. Isoforms of dystrophin are expressed in various forms in different organs, e.g., skeletal muscles, cardiac muscle and the brain. Even if DMD patients achieve expected milestones of motor development, they experience progressive loss of muscular strength. First symptoms of muscle weakness are normally appearing from 18 months to 3 years. Muscle weakness is proximal announced and slowly progressive. Loss of gate without therapeutic support in 9–12 years of age followed by scoliosis, involvement of cardiac muscles. Affection of the respiratory system has a life-limiting effect (Spuler and von Moers, 2004). Between age 12 and 18, this progressive muscular weakening extends to the heart and respiratory system, to the point of provoking premature death (Schaaf and Zschocke, 2018).

Dystrophin is not only present in muscle cells but also plays an important role in brain development (see below). Indeed, deficient production of dystrophin isoforms has severe consequences in different areas of cognitive ability (Taylor et al., 2010), for example in short-term memory capacity (D'Angelo and Bresolin, 2006). Today there is no complete cure for DMD: Indeed, only motor aspects and not cognitive and social functioning can be improved by optimal medical care (e.g., through physiotherapy).

### Important Similarities and Differences Between SMA and DMD

In the above sections we described the genetic conditions for motor impairment in SMA and DMD. Indeed, both children with SMA and DMD (hereafter SMAs and DMDs, respectively) suffer from progressive weakness in the proximal muscles, resulting in the inability to explore the environment without assistive equipment, e.g., wheelchairs or help from caregivers. Additionally, in both disease groups different medical treatments and therapies might impact cognitive functions.

Although DMD and SMA patients both have severe motor impairments, their cognitive and psychosocial functioning differs. Consider first DMDs, where the lack of dystrophin in the hippocampus and the cerebellum (connected with the frontal brain regions) is linked to impaired memory, automatisation, language (for comparison of verbal IQ in SMAs and DMDs, see Billard et al., 1998) and executive functions (Donders and Taneja, 2009; for general cognitive ability, see Cotton et al., 2001; Banihani et al., 2015; Latimer et al., 2017). This particular genetic pattern is aggravated by negative responses from their environment: DMDs accomplish the motor milestones of the first years of life before gradually experiencing the loss of abilities with increasing feelings of helplessness. The combination of genetic factors that impair motor development and executive cognitive functioning as well as experience-dependent psychological factors result in socio-emotional and behavioral problems (Banihani et al., 2015). Comorbid anxiety, obsessive compulsive disorders as well as Attention Deficit Hyperactivity Disorder and even autism spectrum disorders have been reported. ADHD has been found in about 30% of DMDs, leading to severe learning difficulties and problems in social interaction (Pane et al., 2012). In addition, DMDs often suffer from learning disabilities, such as dyslexia and dyscalculia (Hendriksen and Vles, 2006; for an overview, consult Hendriksen et al., 2011).

Consider now SMAs, where cognitive research supports the conclusion that only their motor ability is selectively compromised. Despite severe impairments in motor development, not even their spatial cognition is affected: Indeed, in a three-location-search-task young SMAs (30 months) made fewer mistakes than healthy and age-matched controls (Rivière and Lécuyer, 2002). SMAs outperformed their healthy peers also in language acquisition (Sieratzki and Woll, 2002; Rivière et al., 2009). von Gontard et al. (2002) investigated cognitive abilities in SMA patients between 6 and 18 years by using different test batteries, excluding subtests with motor components. In contrast to DMDs, they do not experience the loss of their motor capacity. Performance above average in older SMAs (over the age of 11) has been interpreted as a strategy to compensate impaired motor ability through advanced creativity and knowledge that is developing over childhood (von Gontard et al., 2002). SMAs use their high social, emotional and cognitive skills to coordinate care givers towards their individual needs.

### Embodied Cognition: The Role of Body-Environment Interactions in Cognition

We now turn to the theoretical motivation for our specific research hypotheses. The recently influential embodied perspective on human cognition rejects the traditional view of the human mind, according to which all our knowledge is mentally represented by amodal symbols contained in semantic memory. On the contrary, it gives the body a crucial role by arguing that cognition uses multi-modal sensory-motor representations originating from the interaction of the organism with its surrounding environment (Barsalou, 1999; Matheson and Barsalou, 2018; for recent reviews, see Fischer and Coello, 2016; Newen et al., 2018). According to embodied cognition, the individual, through action, perception and introspection, acquires concepts which, in turn, are stored in multi-modal memories. These memories are re-activated by simulating selective parts of the acquisition process. As a consequence, even higher-level cognitive processes, such as abstract reasoning or mental arithmetic, rely on involvement of lower-level processes, such as perception and action (e.g., Fischer and Shaki, 2018; Witt, 2018). For example, finger counting is a near-universal mode of acquisition of number concepts that shapes even adult numerical cognition (e.g., Sixtus et al., 2017, 2018).

Following the influential *mental number line* account (Restle, 1970; Dehaene and Cohen, 1995), numbers are represented in the mind as a quantity, based on a spatialized magnitude code. This representation is usually described as continuous, analog, format-independent, and oriented from left to right (in Western cultures; Shaki et al., 2009). Studies on numerical cognition support the hypothesis of a mental number line, revealing the presence of *spatial-numerical associations* (SNAs) and suggesting that spatial processing is crucially involved in numerical cognition. Studies on healthy participants have shown a systematic association between small numbers and left-sided response and between large numbers and right-sided response (Dehaene et al., 1993). Across cultures people represent numbers both on horizontal and vertical mental number lines (Winter et al., 2015).

Embracing the principles of embodied cognition, Fischer (2012) suggests that the spatialization tendency underlying SNAs is not entirely automatic and universal but can be influenced by interactions between the individual and the environment during development. Applying a hierarchical interpretation to SNAs, Fischer (2012) classified them depending on their *grounded aspects* (derived from physical properties present in the real world and universally shared), *embodied aspects* (developed from early experiences of individual-environment interaction and linked to sensory and motor constraints imposed by the body) and *situated aspects* (determined by current contextual constraints). According to this hierarchical view, grounded factors such as the gravity law and the fact that different objects cannot occupy the same space at the same time, explain vertical associations of magnitude, such as MORE IS UP and LESS IS DOWN. Embodied factors such as the habitual use of fingers during counting explain horizontal associations of magnitude with LESS IS LEFT and MORE IS RIGHT*, also* depending on peculiarities of body-context interactions and culture. Finally, situated factors, such as our body postures or temporary characteristics of the surrounding environment, account for the flexibility of the association between space and magnitude (Fischer and Shaki, 2018).

### The Current Study

From the embodied cognition perspective, the fact that children affected by severe motor impairment experience different body-context interactions throughout their lives, should result in different cognitive profiles compared to healthy children. Here, we aim to document such impact of limited sensorimotor experiences on cognitive abilities in the specific context of numerical cognition. Numbers exemplify a domain of knowledge that is highly relevant for everyday life, well defined, easy to activate and involving high-level cognitive processes. Consistent with an embodied approach to cognition, sensory and motor associations systematically emerge during the activation of number concepts (Lindemann et al., 2007; Cohen Kadosh et al., 2008; Fabbri et al., 2012). Therefore, lack of motor activity should result in specific cognitive signatures when thinking about numbers.

This prediction led us to examine whether and how sensory and motor constraints present at an early age might influence numerical cognition. To do so, we used the *Random Number Generation* (RNG) task, a cognitive task first developed to assess executive functions (see, Brugger, 1997 for a review and methodological considerations) and more recently become a benchmark test for the investigation of SNAs (Loetscher et al., 2008; Winter and Matlock, 2013; Göbel et al., 2015; Sosson et al., 2018).

Among the first to use RNG in the field of numerical cognition were Loetscher and Brugger (2007). Specifically, they asked healthy adults to randomly generate numbers between 1 and 6 by imagining to throw a die. Several signatures of numerical cognition emerged: First is a small number bias, defined by the tendency to over-generate the first half, i.e. the smaller numbers of the specified range; whereas a large number bias is defined by the over-generation of the second half, i.e. larger numbers of the specified range. The authors found a significant *small number bias*, defined as the tendency to generate more numbers in the range between 1 and 3 rather than in the range between 4 and 6. Later, Towse et al. (2014) explored number processing in children of different age groups with a larger numerical range (from 1 to 10). In contrast to adults, children reported a *large number bias*, defined as the tendency to generate more numbers in the range between 6 and 10 rather than in the range between 1 and 5. Moreover, differences between age groups indicate that the processing of numbers changes in the course of development. As a second signature of numerical cognition, Towse et al. (2014) formulated a first number bias when analyzing number generation data of very young children. This bias is defined as the tendency to over-generate the very first numbers of the specified range. In association with the large number bias, very young children showed an over-generation of numbers 1, 2 and 3, explained by over-representation of these numbers at an early age (Towse et al., 2014).

Loetscher et al. (2008) assessed the influence of body postures by introducing horizontal head movements into the RNG task. Adult participants were asked to randomly produce numbers from 1 to 30 while continuously moving the head along the horizontal axis (from left to right and from right to left). Winter and Matlock (2013) further enriched the paradigm by adding head movements along the vertical axis. The results revealed the presence of numerical biases along both axes: Participants produced larger numbers after moving their head right (up) than after moving their head left (down). Interestingly, this pattern was stronger in the vertical condition, indicative of a more robust vertical mental number line and in line with the hypothesis of a congruent relation between the situated postural influence and the influence of grounded or physical properties of the world on cognition (Fischer, 2012).

More recently, researchers started using the same paradigm to assess SNAs and their developmental changes in healthy children. In light of different SNAs reported in adults depending on culture (Shaki et al., 2009; see Göbel et al., 2011, for a review), Göbel et al. (2015) compared the influence of reading habits of British and Arabic participants on SNAs in adults and children: participants were invited to randomly produce numbers from 1 to 50, while resting either on their right or on their left side. In general, adult participants generated on average larger numbers than children, which is contrary to the established small number bias in adults. The researchers explained this opposite pattern in light of the different numerical range used in the study: indeed, with numbers from 1 to 50, the small number bias is computed on the range from 1 to 25; these numbers might be more strongly represented in children than higher numbers. Importantly, regardless of age group, body position had an effect on the mean of the generated numbers and this effect depended on the participants' habitual reading direction.

While the above studies investigated SNAs in paradigms where numbers were produced while orienting towards a particular side of space, Sosson et al. (2018) compared average of random number generated by adults and children whenever their head reached the central position during horizontally alternating movements. The results revealed significant biases only in adults, highlighting that the amount of sensorimotor experience, or the ability to predict movement outcomes, might influence the strength of SNAs.

To date, SNAs have never been explored in children with neuromuscular diseases. In this study, by using the RNG task we aim to fill this gap in the literature. Moreover, we assess SNAs along both the horizontal and vertical axis in order to explore the role of grounded and embodied factors in number representation of young children with early motor impairment. Young children with DMD reach milestones of early motor development like walking independently (Connolly et al., 2013). Therefore, self-exploration of the environment is equal to healthy children. Following the embodied perspective those early self-exploring experiences are crucial for the development of mental representations (Rakison and Woodward, 2008).

### Hypotheses

Inspired by the embodied cognition perspective and the hierarchical interpretation of SNAs (Fischer, 2012) we formulated the following hypotheses on RNG performance and different SNAs in the two groups:

H1: We assume stronger SNAs in DMDs compared to SMAs because of their ability to explore the environment in early childhood and, respectively, weaker SNAs in SMAs compared to DMDs because of their more limited exploration.
H2: Finally, RNG is a suitable task also for assessment of executive functions and their maturation. Given that executive functions are impaired in children affected by DMD (Donders and Taneja, 2009), we expected less random number sequences in children with DMD compared to children with SMA at a given age.

## Materials and Methods

### Participants

Children from 6 to 12 years diagnosed with either SMA (type 1 or type 2) or with DMD were included into the study. In total 16 children participated in the experiment: eight SMA (three type 1 and five type 2; three females and five males) and eight DMD children (all males). Mean age of SMA children was 7.62 years (SD 1.4) and mean age of children with DMD was 9.37 years (SD 1.7), *t*_(14)_ = 2.190; *p* = 0.046. Participants were recruited from the department of pediatrics of the German Red Cross Hospitals Berlin. Diagnosis of SMA and DMD was based on molecular genetic analysis of each participant, provided by the hospital or patients' families. Only children with SMA type 1 and 2 were included because, as mentioned in the Introduction, children with SMA type 3 do not differ from healthy children in milestones of motor development.

The current study was approved by the Ethics Committee of the University of Potsdam (ref. nr. 78/2020).

### Tasks and Tests

#### Wechsler Intelligence Scale for Children (WISC-V)

To assess general cognitive performance, three subtests of the *WISC-V* (German Version, Petermann, 2017) were selected, based on what we wanted to measure (verbal ability, non-verbal cognitive performance and working memory) and the suitability of tests to the population with neuromuscular diseases (indeed, only tests not including motor components were included). Cognitive performance was measured by providing different items in ascending levels of difficulty. To assess verbal ability, the subtest *Similarities* was used. Two terms were given and the participant had to tell in what regard they were alike (e.g., the words *red* and *green*, where the correct answer is that both are colours). For measuring non-verbal cognitive performance, the subtest *Matrix Reasoning* was taken from the battery. Children had to choose from a range of abstract pictures the one that completes a given matrix. Finally, the subtest *Letter-Number-Sequencing* was considered to assess working memory. The participant repeated a given span of mixed numbers and letters by arranging them in ascending and alphabetical order. Age-related items as well as norms were used to assess the individual *T*-value with normal (age-appropriate) values between *T* = 40 and *T* = 60.

In all participants general cognitive ability was measured using two subtests from the WISC-V, one for verbal ability (Similarities subtest) and one for performance IQ (Matrix Reasoning subtest). Independent sample *t*-tests revealed no differences between groups, either in the Similarities subtest [*t*_(14)_ =1.479; *p* = 0.161] or in the Matrix Reasoning subtest [*t*_(14)_ = 1.805; *p* = 0.093]. The test scores (*T*) of all SMAs in the similarities subtest were in the normal range (43.3 > *T* < 60.0) except for one participant who performed above average (*T* = 73.3). Instead, DMDs' performance ranged from *T* = 33.3 to *T* = 60.0, including two children below average.

Working memory was assessed through the subtest Letter Number Sequencing of the WISC-V. SMAs performed significantly better than DMDs [*t*_(12)_ = 6.847; *p* < 0.001]. Two participants were excluded from the analysis.

#### Adaptive Intelligence Diagnosticum 3rd Edition (AID 3)

The subtest *Applied Computation* from the *AID 3* (Kubinger and Holocher-Ertl, 2014) was administered for measuring arithmetic skills. Using a branched testing approach, each participant was presented with arithmetic problems that were adapted to their individual level. This ensured high motivation throughout the whole testing. Arithmetic problems included a variety of operations (e.g., additions, subtractions, percentage calculations) in text problem format. An example is: “Petra runs over a 10 m long meadow. Once there and once back. Kurt runs twice as far. How many meters has Kurt covered?” (German: “Petra läuft über eine 10 m lange Wiese. Einmal hin und einmal zurück. Kurt läuft doppelt so weit. Wie viele Meter hat Kurt zurückgelegt?”). Age-related norms were used to assess the individual *T*-value.

The independent sample *t*-test comparing both disease groups' arithmetic performance of the Applied Computation subtest of the AID-3 showed that children with SMA performed significantly better (mean T = 55.25) than children with DMD (mean T = 42.75) [*t*_(14)_ = 2.426; *p* < 0.05].

#### Neuropsychologische Testbatterie für Zahlenverarbeitung und Rechnen bei Kindern—Revidierte Fassung (ZAREKI-R)

In the subtest *Number Line* from the *ZAREKI-R* (von Aster et al., 2014), 12 trials allowed assessment of spatial representation of numbers along a vertical number line from 1 to 100. The task consists of two parts: In the first part additional markers partition the visual lines into specific portions while in the second part no markers are provided. Participants marked the position of each called-out number on the vertical number line. Age-related norms were used to assess the individual test value.

The independent sample *t*-test revealed no significant differences between the two groups in both parts of the subtest [part 1: *t*_(12)_ = 0.111; *p* = 0.913; part 2: *t*_(12)_ = −0.379; *p* = 0.711]. Two participants were excluded from the analysis.

Descriptive data of general cognitive abilities are presented in Table 1.

**Table 1:** Descriptives of *R* scores in the baseline condition and general cognitive data.

	**DMD**	**SMA**	***p*** **(t-tests)**
**R Score (baseline)**	8.35%	8.47%	n.s. (*p* = 0.9)
**WISC-V**			
Similarities (verbal IQ)^a^	45.41 (8.90)	52.49 (10.19)	n.s. (*p* = 0.1)
Matrix reasoning (performance IQ)^a^	42.50 (9.36)	52.50 (12.57)	n.s. (*p* = 0.09)
Letter number sequencing (working memory)^a^	30.44 (8.25)	60.57 (8.00)	***p*** **< 0.001**
**AID-3**			
Applied computation^a^	42.75 (7.40)	55.25 (12.56)	***p*** **< 0.05**
**ZAREKI-R**			
Number line (part 1)^b^	89.00 (29.10)	87.29 (28.70)	n.s. (*p* = 0.9)
Number line (part 2)^b^	65.57 (43.41)	73.29 (31.79)	n.s. (*p* = 0.7)

a *results presented as t-value with standard deviation*.

b *results presented as percentile rank with standard deviation*.

*Bold values are indicating significant effects*.

#### Random Number Generation (RNG) Task

Participants generated random numbers between 1 and 10 while moving their head along either the horizontal axis (alternating left-to-right, horizontal condition) or vertical axis (alternating down-to-up, vertical condition) or by keeping the head straight (baseline condition). We considered the following parameters: mean of generated numbers and differences between every generated number and its immediately preceding one, i.e., *first order differences* (FODs). The sign of FODs provides information about direction of movement on the mental number line (positive/negative FODs correspond to ascending/descending tendency), instead, the absolute value of FODs indicates the size of movements on the mental number line. Additionally, a redundancy (*R*) score was computed as an index of randomness (the higher the *R* score, the lower the randomness).

#### Dot Counting Direction Task (DCD)

During the DCD (Shaki et al., 2012; Fischer and Shaki, 2017), participants sequentially count aloud four dots displayed on a DIN A4 sheet horizontally (left-right) or vertically (up-down) in front of them, by pointing on each dot with their finger. This task is useful to assess directional counting habits. The instructions were: “Please count the number of these dots, by pointing at each of them with your finger” (in German: “Bitte zähle die Punkte nacheinander laut, indem Du mit Deinem Finger auf jeden Punkt zeigst”). We recorded the order of pointing.

### Procedure

Due to COVID-19 restrictions, all participants were tested via the online video platform ZOOM. After receiving informed consents from the parents via mail the ZOOM link was sent via mail. To avoid any numerical and lateral cues during the test session, participants were asked to cover the keyboard in front of them and to remove all numerical stimuli (e.g., clocks or pictures) from their surroundings. The experiment was conducted in German language. All participants' parents gave permission to video-record the testing session for further offline evaluations.

Firstly, all participants completed the RNG task. We used a mixed design with Head position (left vs. right vs. up vs. down vs. straight) as within-subjects factor and Disease (SMA vs. DMD) as between-subject factor. For each participant, there were four experimental conditions (left vs. right (horizontal) vs. up vs. down (vertical) as starting position of the head) and one baseline condition (straight). The axes (horizontal vs. vertical vs. straight) were presented in a blocked order while the head positions alternated continuously within each block, along either the horizontal or vertical axis. The experimental conditions (horizontal and vertical) were counterbalanced across participants while the baseline condition was always administered at the end.

Participants were asked to generate numbers between 1 and 10 as randomly as possible while moving their head from left to right (experimental condition horizontal) or up to down (experimental condition vertical). Importantly, the instructions clarified that numbers were only to be stated once the participant's head was in the most extreme (yet still comfortable) position, not before. To facilitate understanding of randomness a ten-sided die was used. A stencil of such a die was sent to the families together with informed consents and the instruction to build it before testing. In our experience, providing participants with this manipulable object facilitates the understanding of the RNG task. The following instruction was used: “Imagine you were a 10-sided-die rolling from one side to another. While rolling please say the sentence “*The die is showing number…* and then please call out a number between 1 and 10.” (In German: “Der Würfel zeigt die Nummer.”). Participants were instructed to keep their eyes closed during the complete task. Due to differences in the degree of motor impairment, participants determined their own speed of responding.

After the horizontal and vertical block of the RNG task, the horizontal and vertical version of the DCD was administered. The ZAREKI-R number line subtest followed afterwards. Depending on the fatigue of each participant, assessment of general cognitive abilities was conducted either immediately following or in a separate second session that took place up to 7 days later.

### Data Preparation

First of all, omissions, errors (all not numerical oral responses, e.g., letters) and numbers out of the instructed range were detected for each condition and group. Both SMA and DMD groups performed only one omission in the right-side head starting condition and one error in the baseline (head straight) condition. In the SMA group there were four numbers out of the range each in the up and in the down condition, respectively. The DMD group generated numbers out of the range in all conditions: one in the down, three in the up, four in the left, two in the right and three in the baseline condition.

No participant was excluded from the analysis. One participant did not follow the instruction completely and changed the sentence preceding the RNG responses (“*The die is showing number…*”) to “*aloha*”. Nevertheless, he did not vary this sentence during the trials and his performance did not deviate from that of the others. For these reasons, that participant was also included.

Errors, omissions and numbers out of the range were removed together with their preceding and succeeding numbers (see Sosson et al., 2018, for methodological considerations).

### Data Analyses

Firstly, we compared the means of the generated numbers and FODs for each participant individually in all five head positions to detect descriptive group differences followed by statistical analysis using multilevel modelling with the generated number and FODs in each head position as a function of group (DMD vs. SMA). We decided to take that approach to enrich the information gained from the data by emphasizing individual differences. Because of the more fine-grained depiction of the data on the level of head position (left, right, up, down, straight) we can increase power compared to averaging the data as would be done in parametric statistics.

In addition, *R* scores were computed using the software developed by Towse and Neil (1998). To explore differences between the patient groups and normally developing children, baseline *R* scores of DMDs and SMAs were compared to the results reported by Towse and McIachlan (1999) and *R* scores in the horizontal condition were compared to the more recent data by Sosson et al. (2018). Finally, for the two groups, indices for general cognitive ability and arithmetic skills were compared via *t*-tests.

## Results

### Spatial Numerical Association (Hypothesis 1)

We will start the analysis by taking a closer look at the mean differences between head positions within and between the two groups. Next, we set up multi-level models (random intercepts with responses nested in persons) to test the corresponding hypotheses. In these models we predicted the generated number by head position (model for horizontal positions: left vs. right; model for vertical positions: up vs. down), group (SMA vs. DMD), and the interaction head position × group (see equation 1). Head position and group were dummy coded with left (model 1) and down (model 2) as the reference category for head position and DMD the reference category for group. All estimators were optimized for minimizing the restricted maximum likelihood. Estimation weights are reported non-standardized.

(1) $$\begin{array}{l} Generatednumber_{ij}=\beta 0_{j}+\beta 1headposition_{ij}\\ \;+\beta 2headposition_{ij}\times groupj+\epsilon_{ij}.\\ \;\beta 0_{j}=\gamma 00+\gamma 01group_{\text{1}j}+u0_{j}\\ \;i:=trial\\ \;j:\;=person \end{array}$$

The analyses were conducted with the R package lme4 (Bates et al., 2015).

Additionally, FODs were taken instead of the magnitude of the generated number as the dependent variable, we also conducted all analyses and models with FODs. The results are nearly identical and will be reported in the Tables 5, 6 in Supplementary Material of this paper to avoid redundancy.

#### Description of the Means

Following Sosson et al. (2018), we start by reporting the mean of generated numbers and FODs, separate for all head positions (left, right, down, up, straight) and groups (SMA, DMD), present in Table 2.

**Table 2:** Descriptive information for all head positions in both groups.

**Group**	**Head position**	**Mean number (sd)**	**Mean FOD (sd)**	**Total of omissions**	**Total of errors**	**Total** ***n*** **out**
DMD	Left	5.80 (2.94)	−0.07 (3.49)	0	0	4
	Right	5.99 (2.79)	0.08 (3.86)	1	0	2
	Down	5.28 (2.83)	−0.33 (3.80)	0	0	1
	Up	5.57 (3.04)	0.27 (3.72)	0	0	3
	Straight	5.49 (2.87)	0.05 (3.62)	0	1	3
SMA	Left	5.53 (2.98)	0.21 (4.14)	0	0	0
	Right	5.54 (3.04)	−0.10 (4.19)	1	0	0
	Down	6.10 (2.83)	0.69 (3.82)	0	0	4
	Up	5.42 (3.07)	−0.75 (4.36)	0	0	4
	Straight	5.61 (3.02)	0.09 (4.32)	0	1	0

In the horizontal condition the mean of generated numbers is higher in children with SMA than DMD. Additionally, in children with DMD the mean of generated numbers is higher in the right-head (*M* = 5.99; *SD* = 2.79) than in the left-head position (*M* = 5.80; *SD* = 2.94), whereas in children with SMA it is similar in the left (*M* = 5.53; *SD* = 2.98)- and right-head position (*M*= 5.54; *SD* = 3.04). In the vertical condition children with SMA produced fewer small numbers when turning the head down (*M* = 6.1; *SD* = 2.83) compared to up (*M* = 5.54; *SD* = 3.07), whereas children with DMD reported the reversed pattern. They produced more small numbers in the down (*M* = 5.28; *SD* = 2.83)- and larger numbers in the up-head position (*M* = 5.57; *SD* = 3.04).

The following pattern was found in FODs: In the horizontal condition, children with SMA produced more ascending sequences when turning their head left (*M* = 0.21; *SD* = 4.41) than right (*M* = −0.10; *SD* = 3.49), while the opposite pattern was observed in DMD children with more ascending sequences generated during right (*M* = 0.08; *SD* =3.86) rather than left (*M* = −0.07; *SD* = 3.49) head position. In the vertical condition children with SMA tended to generate more ascending sequences (represented by positive FODs) when turning the head down (*M* = 0.69; *SD* = 3.82) rather than up (*M* = −0.75; *SD* = 4.36). A reversed pattern was observed in DMD children, who performed more ascending steps when turning the head down (*M* = −0.33; *SD* = 3.80) rather than up (*M* = 0.27; *SD* = 3.72). The FODs measures corroborated tendencies revealed by the previously considered mean of generated numbers.

#### Hypothesis Testing

Table 3 shows the estimations for the multi-level model of the horizontal condition. Firstly, we included *head position* as a predictor (model 1) and then the *group and head position* × *group* (model 2). Neither *head position* (*B* = 011, *p* = 0.616), *group* (*B* = −0.26, *p* = 0.450) nor the interaction *head position* × *group* (*B* = −0.19, *p* = 0.686) significantly impacted the magnitude of the generated numbers.

**Table 3:** Multilevel model for mean of generated numbers in left-right head movements (horizontal).

	**Model 1**				**Model 2**			
***Predictors***	***B***	***se***	***t***	***p***	***B***	***se***	***t***	***p***
(Intercept)	5.66	0.18	31.94	**<0.001**	5.80	0.25	23.02	**<0.001**
Head position (right)	0.11	0.23	0.50	0.616	0.20	0.33	0.60	0.545
Group (SMA)					−0.26	0.35	−0.76	0.450
Head position (right) × Group (SMA)					−0.19	0.46	−0.40	0.686
**Random effects**								
σ^2^	8.56				8.57			
τ_00_	0.07 _ID_				0.05 _ID_			
ICC	0.01				0.01			
N	16 _ID_				16 _ID_			
Observations	657				657			
Marginal *R*^2^/Conditional *R*^2^	0.000/0.008				0.004/0.010			

*Bold values are indicating significant effects*.

In the vertical condition (up-down head movements, Table 4) we, again, did not find a significant effect of *head position* (B = −0.18, *p* = 0.435). That is, *head position* had no impact on the generated number for the DMD group. But the generated numbers were significantly higher for the *SMA group* (B = 0.83, *p* < 0.05) and the *SMA group* also had a significantly stronger effect of the *head position* compared to the DMD group (B = −0.97, *p* < 0.05).

**Table 4:** Multilevel model for the generated numbers in up-down head movements (vertical).

	**Model 1**				**Model 2**			
**Predictors**	***B***	***se***	***t***	***p***	***B***	***se***	***t***	***P***
(Intercept)	5.67	0.18	31.99	**<0.001**	5.28	0.24	21.66	**<0.001**
Head position (up)	−0.18	0.23	−0.78	0.435	0.29	0.33	0.89	0.374
Group (SMA)					0.83	0.35	2.36	**0.018**
Head position (up) × Group (SMA)					−0.97	0.47	−2.08	**0.037**
**Random effects**								
σ^2^	8.65				8.60			
τ_00_	0.07 _ID_				0.06 _ID_			
ICC	0.01				0.01			
N	16 _ID_				16 _ID_			
Observations	636				636			
Marginal *R*^2^/Conditional *R*^2^	0.001/0.009				0.011/0.018			

*Bold values are indicating significant effects*.

### Randomness Performance (Hypothesis 2)

The redundancy scores (*R*) in both groups were computed separately for both head positions along the vertical and horizontal axis and the baseline (straight head position) by using the software from Towse and Neil (1998). *R* scores can range between 0 and 100% where 0% means complete randomness in number generation and 100% results from a completely predictable sequence. The observed average *R* scores were 8.47% (range 1.14–16.66%) for SMA children and 8.35% (range 1.78–17.47%) for DMD children. A two-tailed independent *t*-test limited to the straight head condition showed no significant difference between SMAs and DMD's *R* scores [*t*_(14)_ = −0.042; *p* = 0.967, Table 1].

Next, we compared the performance of our sample against that of healthy children by referring to published *R* values in comparable conditions. Towse and McIachlan (1999) assessed the RNG task with children of different age groups by keeping the head straight. Two-tailed single sample *t*-tests for each group in the straight head condition were computed against the reference value. We compared the *R* values of the SMA group against *R* = 2.29%, a value that has been reported for a sample of approximately 8 years olds. We obtained a significant deviation from those reference values of healthy children in SMAs [*t*_(7)_ = 3.082; *p* = 0.018; *R* = 8.47%]. The score of *R* = 8.35 % for our DMDs was compared against the reference value of *R* = 4.18 % of the older subsample (average age 10 years). No significant group difference was revealed [*t*_(7)_ = 1.899; *p* = 0.099; *R* = 8.35%]. Sosson et al. (2018) introduced left/right head movements to the RNG task. We compared performance of our two groups in the horizontal condition with their *R* score of 10.64% for healthy children (age range 7.8–11.9 years). A two-tailed single sample *t*-test for each group in the horizontal condition was computed. *t*-tests revealed significant deviations from reference values of healthy children in SMAs [*t*_(7)_ = −7.128; *p* < 0.001; *R* = 3.65%] as well as in DMDs [*t*_(7)_ = −5.650; *p* < 0.005; *R* = 4.49%].

### Exploratory Analysis: Dot Counting Task

All participants counted the four dots successively from one side to the other. All SMAs counted from left to right except for one participant who counted from right to left. Interestingly, this child also generated on average larger random numbers with left-turned head (RNG *M* = 6.27) than with right-turned head (RNG *M* = 5.67). Three of eight DMD participants counted from right to left (left-turned head: RNG *M* = 5.50; right-turned head: RNG *M* = 5.59).

On the vertical axis counting habits were the following: For SMAs again only one participant counted from down (RNG *M* = 6.00) to up (RNG *M* = 5.60). Among DMDs two of eight children counted from down to up (down-turned head: RNG *M* = 5.31; up-turned head: RNG *R* = 6.55).

## Discussion

Inspired by an embodied approach to cognition, the current study aims to investigate number processing and higher order cognitive abilities in children with rare neuromuscular diseases. We tested eight SMA and eight DMD children between 6 and 12 years in a Random Number Generation task. Since it is a suitable task for numerical cognition as well as for executive functions, we derived two distinct predictions. Below, we discuss the findings following our hypotheses: Hypothesis 1 is related to spatial numerical associations, hypothesis 2 is related to randomness, indicative of inhibitory control.

### Spatial-Numerical Associations (Hypothesis 1)

For the first time, spatial-numerical associations (SNAs) were assessed in children with motor impairments, examining their SNAs along both the horizontal and the vertical axis. We hypothesized, that SNAs would differ between children with SMA and DMD due to differences in the extent of self-exploration in early childhood. Although patterns of SNAs in children with SMA were atypical, they performed above average in general cognitive and arithmetic tasks. Participants with DMD, on the other hand, revealed SNAs comparable to those expected of healthy children but showed weaker performance in arithmetic and in general cognitive ability and executive functioning.

Even though no systematic difference in the counting direction was found in the DCD between DMDs and SMAs, participants with DMD had a tendency to generate larger/smaller numbers after turning their head rightward/leftward, but the ones with SMA did not present a preference in the left-right direction. This may perhaps be related to the severe and early handicap in changing their postural positions including an inability to take an upright body posture as well as to a less mature development of functional laterality in the significantly younger SMAs.

Individual differences within as well as between the two groups were observed for horizontal SNAs and can be interpreted following the hierarchical view on embodied cognition (Fischer, 2012). The differences between the groups can be explained by distinct motor experiences in the course of the two diseases, i.e., on the embodied level. Additionally, in both groups for some participants, testing was only possible with a caregiver next to them. The lateral presence of another person might anchor the participant and therefore has an impact on number generation, i.e., on the situational level. Three out of seven participants with a caregiver sitting next to them did show reversed or no horizontal SNAs.

Concerning next the vertical dimension, DMDs showed patterns comparable to healthy developing children by performing more descending/ascending steps in the downward/upward head orientation, respectively. In contrast, SMAs revealed a reversed tendency. How to explain this opposite trend? From an embodied point of view, early sensorimotor experience is crucial for developing mental representations. First, disease onset in DMD is around age 6–7 which indicates that several milestones of motor development are reached before outbreak of the disease and loss of muscle strength. In contrast, SMA children do not typically acquire motor skills such as sitting or standing autonomously. Therefore, compared to SMAs, experiences of DMDs do not differ that much from healthy developing children in those first and important years of life. Their results in this task of embodied number processing can therefore be equal to the ones of healthy children.

An additional interpretation of the unique pattern of RNG performance in children with SMA along the vertical axis pertains to a different internalization of universal concepts related to physical properties of the world. Indeed, due to their inability to reach low or high points in space, SMAs could be more sensitive to an object's weight. From an early age, they start experiencing the weight of their limbs and as they grow and become taller, their body gets heavier without gaining more muscle strength like healthy children. Associations might be built between more weight (i.e., heavy) and downward space and less weight (i.e., light) and upward space, respectively. The vertical spatial representation of weight is a rarely studied but upcoming topic. Across domains “more” is associated with “up” and “less” is associated with “down”. According to the hierarchical view on embodied cognition, weight could play a distinct role on the situated level: A recent study by Vicovaro and Dalmaso (2020) found a reversed SNARC-like effect when participants were directly instructed towards the weight of objects. They responded faster with a downward button for higher weights and with an upward button for lower weights. This effect was only found when weight was task relevant. This finding could support our interpretation: The weight of an object is of specific valence for children with SMA.

### RNG and Randomness (Hypothesis 2)

We measured the randomness of our participants with SMA and DMD by computing the redundancy score *R*. Low redundancy equals high randomness in number generation. Impairments in executive functions are linked to the inability to inhibit certain tendencies in number production (see Brugger, 1997; Peters et al., 2007). Research with DMD children revealed impairment in executive functions (Donders and Taneja, 2009). Therefore, we hypothesized that performance of children with DMD would have been less random than performance of participants with SMA. However, both groups' redundancy did not differ significantly in the current study. Developmental improvements in performance were established in studies on random generation with children without motor impairment (Towse and McIachlan, 1999). Additionally, *R* scores of both groups differed significantly from *R* scores of healthy children on the horizontal axis (Sosson et al., 2018) but only for SMAs when excluding the spatial component from the task (Towse and McIachlan, 1999). It is worth noticing that, since in Sosson et al. (2018) the numerical range was larger than the one used in our and in the Towse and McIachlan (1999) study and since increase of numerical range reduces the randomness (Towse and McIachlan, 1999), the comparison with the index computed by Sosson et al. (2018) should be considered with precautions. Nevertheless, higher age in DMDs contributes to them performing more similar to healthy controls as executive functions as well as motor experience evolve with age.

### Cognitive Profile

Since SMA and DMD are associated with several differences in general cognitive abilities, we assessed the cognitive profile of the two groups by administering subtests from established test batteries (WISC-V and AID-3). Verbal and non-verbal performance was assessed by two subtests from the WISC-V. It was expected that children with SMA would outperform children with DMD in verbal IQ whereas performance related assessment would not show any differences. The current study did not find significant distinctions in verbal ability between the two groups, even though tendentially SMA children did outperform DMD boys. The lack of a significant difference in the predicted direction might also reflect the reliable age advantage of DMD children in our study.

### Limitations and Outlook

The current study fell right into the COVID19-crisis. Testing participants in conditions affecting the respiratory system made avoidance of any infection risk even more important. We decided to test the participants online via the platform ZOOM. Despite the advantage of being more flexible in scheduling the appointments and incurring less effort for the families, some limitations had to be accepted.

In light of contextual influences on number processing (Fischer et al., 2010), RNG performance may be sensitive to task-irrelevant numerical cues in participants' visual field. In our on-line study, it was not possible to sufficiently control for such visual cues, as well as for auditory noise coming from other persons or even construction work. Setting up and monitoring the technical equipment required care givers being close to the participants. Exploring the influence of lateralized cues on RNG performed by SMAs and DMDs would identify the weights of contextual factors (Fischer, 2012).

One potential limitation of the current study is the missing age matched control group of healthy children. Here, we compared performance of children with rare neuromuscular diseases to reported results in former studies with healthy and normally developing children by Towse and McIachlan (1999) and Sosson et al. (2018). Future research should provide a direct comparison of children with SMA and DMD with age matched healthy controls.

Finally, the rarity of SMA and DMD led to rather small samples and contributed to the significant age difference between the two groups. Bigger sample sizes in future research should compare groups with different degrees of motor impairment and finger dexterity (Guedin et al., 2018).

## Conclusion

To summarize, DMDs, unlike SMAs, seem to have both a horizontal and a vertical mental number line, similar to healthy children. In other words, DMDs' performance supports the hypothesis that, due to equal sensorimotor experience, the mental representation of numbers of DMDs is comparable to that of healthy developing children. The observed discrepancy between typically developing SNAs and atypically (weak) developing math abilities in DMDs is likely due to deficits in the development of supportive domain general cognitive functions, like executive and attentional regulation, as well as due to psychological coping difficulties with gradual loss of motor ability. For SMAs it seems unlikely that the observed atypical SNAs are at all disadvantageous for the development of their high math abilities. They seem to be able to spatially mentalize numbers albeit their SNAs develop atypically in spatial direction and strength.

In general, this supports the view that typical as well as atypical development of numerical cognition cannot be predicted by single factor models (i.e., core ability / deficit) but rather by multiple factor models that cover a wide range of biological as well as environmental individual differences (Kaufmann et al., 2013). Moreover, our results support and further develop the hierarchical view by Fischer (2012), demonstrating how the properties of the body inhabiting the brain and the development of compensatory skills are determinant in the use of different spatial information (derived from physical properties or acquired through daily activities) for number representation and processing.

## Data Availability Statement

The raw data supporting the conclusions of this article will be made available by the authors, without undue reservation.

## Ethics Statement

The studies involving human participants were reviewed and approved by Ethics Committee of the University of Potsdam (ref. nr. 78/2020). Written informed consent to participate in this study was provided by the participants' legal guardian/next of kin.

## Author Contributions

HS, AF, and MF: conceived and designed the experiments. HS: collected data. HS, AF, and JW: analyzed the data. HS and AF: wrote the paper. AM: advised in medical terminology and provided contact to participants. JW, MA, and MF: advised in methodological aspects. MF, AM, JW, and MA: proofread the manuscript. All authors contributed to the article and approved the submitted version.

## Conflict of Interest

The authors declare that the research was conducted in the absence of any commercial or financial relationships that could be construed as a potential conflict of interest.

## Publisher's Note

All claims expressed in this article are solely those of the authors and do not necessarily represent those of their affiliated organizations, or those of the publisher, the editors and the reviewers. Any product that may be evaluated in this article, or claim that may be made by its manufacturer, is not guaranteed or endorsed by the publisher.

## Abbreviations

SMA: spinal muscular atrophy
DMD: Duchenne muscular dystrophy
SMAs: children with SMA
DMDs: children with DMD
SNAs: spatial numerical associations
RNG: random number generation
FODs: first order differences.
