# Supplementary material for: Neuromuscular Diseases Affect Number Representation and Processing: An Exploratory Study
Source: Front Psychol. 2021 Sep 6;12:697881. doi: 10.3389/fpsyg.2021.697881 (PMC8450493; doi:10.3389/fpsyg.2021.697881)
Supplement: Supplementary file 1 [file Data_Sheet_1.docx]

# Supplementary Material

**Table 5** Multilevel model for mean of FODs in left-right head movements (horizontal)

|  | **Model 1** | | | | **Model 2** | | | |
| --- | --- | --- | --- | --- | --- | --- | --- | --- |
| *Predictors* | *B* | *se* | *t* | *p* | *B* | *se* | *t* | *p* |
| (Intercept) | 0.08 | 0.22 | 0.35 | 0.728 | -0.07 | 0.32 | -0.21 | 0.834 |
| Head position (right) | -0.09 | 0.31 | -0.28 | 0.779 | 0.14 | 0.44 | 0.33 | 0.745 |
| Group (SMA) |  |  |  |  | 0.28 | 0.45 | 0.62 | 0.533 |
| Head position (right) x  Group (SMA) |  |  |  |  | -0.45 | 0.62 | -0.73 | 0.466 |
| **Random Effects** | | | | | | | | |
| σ^2^ | 15.49 | | | | 15.52 | | | |
| τ_00_ | 0.00 _ID_ | | | | 0.00 _ID_ | | | |
| N | 16 _ID_ | | | | 16 _ID_ | | | |
| Observations | 642 | | | | 642 | | | |
| Marginal R^2^ / Conditional R^2^ | 0.000 / NA | | | | 0.001 / NA | | | |

**Table 6** Multilevel model for mean of FODs in up-down head movements (vertical)

|  | **Model 1** | | | | **Model 2** | | | |
| --- | --- | --- | --- | --- | --- | --- | --- | --- |
| *Predictors* | *B* | *se* | *t* | *p* | *B* | *se* | *t* | *p* |
| (Intercept) | 0.17 | 0.22 | 0.74 | 0.460 | -0.33 | 0.31 | -1.05 | 0.292 |
| Head position (up) | -0.42 | 0.32 | -1.32 | 0.186 | 0.59 | 0.45 | 1.32 | 0.187 |
| Group (SMA) |  |  |  |  | 1.02 | 0.44 | 2.29 | **0.022** |
| Head position (up) x Group (SMA) |  |  |  |  | -2.03 | 0.63 | -3.20 | **0.001** |
| **Random Effects** | | | | | | | | |
| σ^2^ | 8.65 | | | | 8.60 | | | |
| τ_00_ | 0.07 _ID_ | | | | 0.06 _ID_ | | | |
| ICC | 0.01 | | | | 0.01 | | | |
| N | 16 _ID_ | | | | 16 _ID_ | | | |
| Observations | 636 | | | | 636 | | | |
| Marginal R^2^ / Conditional R^2^ | 0.001 / 0.009 | | | | 0.011 / 0.018 | | | |

**R Code for calculated multilevel modeling**

Multilevel model for FOD in SMA and DMD:

model1 <- lmer(FOD ~ head + (1|ID), data = dat_tmp)

model2 <- lmer(FOD ~ head + (1|ID) + head*group, data = dat_tmp)

Multilevel model for generated number in SMA and DMD:

model1 <- lmer(number ~ head + (1|ID), data = dat_tmp)

model2 <- lmer(number ~ head + (1|ID) + head*group, data = dat_tmp)
